# Transitivity Violations Undermine Rating Scales in Motivation Research

**DOI:** 10.3389/fpsyg.2021.632991

**Published:** 2021-09-30

**Authors:** Yulia Tyumeneva, Kseniya Vergeles

**Affiliations:** ^1^Institute of Education, National Research University Higher School of Economics, Moscow, Russia; ^2^Center for Cognition and Communication, Pushkin State Russian Language Institute, Moscow, Russia

**Keywords:** measurement, ordinal structure, transitivity, motivation, rating scale

## Abstract

Measures of psychological attributes, such as motivation, typically involve rating scales, assuming that an attribute can be ordered. If an attribute has an ordinal structure, its levels stand in ordinal relations to one another, and these must be transitive. We tested if transitivity is preserved when people compare different motives in terms of their importance to learning. We found transitivity violations in both strict (Study 1) and non-strict (Study 2) orderings in about half of the participants. Nevertheless, based on the distribution of such violations, we conclude that an ordinal structure of motivation can be found, but only when levels of motives differ noticeably. As the levels become subjectively similar, transitivity is not preserved, and the ordinal structure cannot be justified even in non-strict ordering. The findings question the mainstream practice of measuring psychological attributes before their structure is properly explored.

## Introduction

Mainstream measures of motivation typically involve ratings scales, assuming that motivation can be ordered by its magnitude. However, the fact that rating scales are technically applied does not mean that the very attribute has an ordinal structure. Based on Hölder’s mathematical axioms for quantity (Hölder, 1901; as cited in Michell and Ernst, 1996), levels of attribute can only define an order if they are related in a transitive, asymmetric, and connective manner. For example, to be transitive, any of the levels of attributes *a*, *b*, and *c*, must satisfy the following condition: if *a>b* and *b>c*, then *a>c*. If the order conditions are not met, then levels of the attribute cannot be ordered, and it cannot be measured on the ordinal scale. Moreover, the ordinal structure is a prerequisite for quantitative structure (Michell, 1999, 2003a, 2012). Hence, not meeting the order conditions automatically makes the attribute immeasurable on the interval scale.

Whether an attribute structure is quantitative (ordinal and additive), ordinal, or we can only correctly judge it as the same or different, is an empirical hypothesis (Michell, 1999, 2003a); it must be tested experimentally before proceeding to measurement. However, the prevailing psychological practice ignores the need to explore the inner nature of psychological attributes and continues imposing a pre-defined structure of the measurement model (such as an equal interval rating scale) on a poorly defined psychological attribute. Repeated critiques remain basically unanswered (Cliff, 1992; Barrett, 2003, 2018; Michell, 2003b, 2008; Trendler, 2013; Uher, 2021).

Regarding motivation, one of the most popular subjects of psychological assessment, rating scales still remain a measure by default, although little or no research has been devoted to examining the structure of motivation. Against this background, we addressed motivation to check the validity of the assumption that motivation has at least an ordinal structure.

## Rating Scales in Measuring Motivation

In quantitative psychology, motivation is usually measured on self-rating scales, focusing on different “types” of motivation. These types may differ with regard to a driving source (intrinsic or extrinsic motivation), a domain of activity (learning, academic, work motivation, etc.), goal orientation (achievement or failure avoidance motivation), and so forth.

Typically, individuals are asked to rate to what extent different events, goals, or feelings drive them to engage in some activity (such as going to university, studying math, etc.). Items presumably reflecting intrinsic motivation are statements such as “I study math because I like it,” “… because I am interested in these topics,” and “… because I want to learn more about it.” Items presumably reflecting extrinsic motivation include “I go to university because it is a prerequisite for business life,” “… because it is necessary at my age,” “… because my parents want me to,” and so on. A Likert-type response scale usually offers five categories graduated from “strongly disagree” to “strongly agree.” The ratings obtained are then transformed into scores that are supposed to reflect an individual level of some specific motivation (e.g., intrinsic or extrinsic, achievement, and learning motivation). Statistics behind score generation can vary, but it overwhelmingly treats ratings as quantitative measures allowing for quantitative conclusions such as between-group differences or factor structure of motivation.

Clearly, a number of assumptions behind this practice remain untested, such as that types of motivation all have an identical structure across individuals and situations, or that the five-point scale isomorphically represents the motivational structure. Here, we address the most basic assumption, which is necessary, but not sufficient, for quantitative measurement: motivation has at least an ordinal structure, that is, levels of motivation can be ordered. Specifically, transitivity between levels of motivation is tested. We check whether people can preserve an order in ratings of motivation types, that is, if they rate motive A higher than motive B, and motive B higher than motive C, then A must be higher than C.

Transitivity (if *a*>*b*>*c*, then *a*>*c*) is a basic condition for ordering, so it must be satisfied if an attribute magnitude is ordinal. Although it has been shown that testing transitivity in people’s preferences is possible (Michell, 1998), the literature provides mostly theoretical discussions of the issue. To the best of our knowledge, only one study has attempted to discover the ordinal structure of an attribute. Morris et al. (2017) checked the non-strict transitivity of neuroticism ratings. In this experiment, each respondent made several pairwise rankings of themselves and other people on items from the “neuroticism scale” (NEO-PI-R). If transitivity had been preserved, then people must have ordered others so that if person A is rated higher than or equal to person B, and person B is higher or equal to person C, then A must be higher than or equal to C. It was found that participants did not preserve order in their ratings in 12–25% of cases, meaning that a basic axiom of ordinal structure is violated. These results undermine the psychological claim about the ordinal structure of personality traits and rating scales as a method to measure them.

## The Current Study

Following Morris et al. (2017), we examined the transitivity of motivation using pairwise comparisons. Nevertheless, the current study differs from Morris et al. (2017) in some important features. First, participants were asked to compare attributes (different motivation types) rather than people. We hypothesized that the high rate of transitivity violations found in Morris’ study may have stemmed from the inconsistency of the images of other people when trying to order these regarding a particular attribute. If so, comparing attributes regarding yourself instead of comparing other people in relation to an attribute could reduce these violations.

The second distinctive feature of the current research is that while Morris et al. (2017) tested weak transitivity (if *a* ⩾ *b* ⩾ *c*, then *a* ⩾ *c*), using comparisons with three possible outcomes: “more,” ““less,” and “equally,” we tested both weak and strict (if *a*>*b*>*c*, then *a*>*c*) transitivity, not using “equally” in the last case. The necessity of the “equally” option is supposed to reduce transitivity violations, which come from the ordering of attributes with subjectively equal magnitudes (Michell, 1998). In the absence of the “equally” option, the closer the intensity of attributes to each other, the higher the chance that they will be ordered erroneously. Hence, we expect the highest transitivity violation rate when attributes have equal magnitude. However, the “equally” option may not really prevent this kind of transitivity violations because the same violations can come from an erroneous classification of magnitudes as equal or not.^1^ In fact, the chances for misclassifications even increase as there are three classes (more/less/equal), instead of two (more/less) with which magnitudes must be matched. Given these alternatives, we tested transitivity violations under both strict (Study 1) and weak (Study 2) conditions.

As three attributes for pairwise comparisons are needed to test transitivity in ordering, we settled on intrinsic, extrinsic, and social types of motivation for learning. The first two types are perhaps the most popular in research. The concept of intrinsic motivation can be traced back to a prominent work by Robert White who put forward “competence motivation” as stemming from a tendency to explore and master the environment without other surplus goals, except pleasure in exercising and developing the “ego function” (White, 1959). “Competence motivation” is intrinsic in the sense that no other incentives are required to engage in activities. It differs from extrinsic motivation, which is oriented to external benefits or caused by external circumstances (Deci and Ryan, 2008). Although more recent theories view intrinsic and extrinsic motivations as segments on a continuum rather than a dichotomy (Ryan and Deci, 2000), the division of these types continues to appear in the research literature. Moreover, these two types are often measured and interpreted as independent factors (Guay et al., 2000; Gordeeva et al., 2014).

The third type of motivation we selected can be termed “social motivation,” that is, seeking interpersonal relationships, engagement in social life, and social support from peers. Although social motivation is not a common motivation type in the literature, some studies have shown that it is an important predictor of engagement in activities, such as learning or work (Geen, 1991; Wentzel, 1996, 1999; Ryan and Patrick, 2001; Garn et al., 2011). Furthermore, social motivation is clearly distinguished from both intrinsic (as inner interest in an activity) and extrinsic (as pressing circumstances or earning incentives) types, allowing comparison according to their magnitude.

## Study 1. Strict Transitivity

In this study, we tested transitivity violations in strict ordering of three motivation types. If these types have an ordinal structure, transitivity in sets of pairwise comparisons will be preserved.

### Method

#### Participants

A total of 275 university students (average age=20.4; *SD*=2.1), who were participating in a larger unrelated survey, completed our questionnaires online. Quality control was carried out and cases with unrealistically quick answers were removed from the analysis. Eventually, 250 cases were included in the analysis.

#### Instruments and Procedure

The statements belonged to either the intrinsic, extrinsic, or social motivation type.

The statements corresponding to intrinsic motivation (I) were as follows:

- I am interested in learning (i1).
- what we study is important for me (i2).
- to know more about what we study (i3).

The statements corresponding to extrinsic motivation (E) were as follows:

- to obtain a diploma (e1).
- otherwise, I will not be able to find a job (e2).
- to worry less about my exams (e3).

The statements corresponding to social motivation (S) were as follows:

- to meet my friends (s1).
- to meet new people (s2).
- because, it has a sense of community (s3).

We paired statements from each motivation type with each statement from other types; statements from the same type were not paired. There were nine IE pairs, nine ES pairs, and nine IS pairs (27 pairs in total) in the questionnaire. For example, three items i1, e1, and s1 were presented in three pairs: (1) i1 vs. e1, (2) e1 vs. s1, and (3) i1 vs. s1 to choose a dominant motive in each pair (see Appendix). These pairs made up a triad i1-e1-s1, which we checked for transitivity. If a triad included motives that cannot be ordered, say, a participant considered I over E, E over S, and S over I in pairwise comparisons, then this triad violated transitivity.

The questionnaire included a brief introduction: “Students may have different interests and motives when going to university.” The purpose of this short survey is to determine the relative weight of the different motives that “drive” you in your learning. Motives are grouped into pairs. Both motives in each pair may be important to you, but you need to choose the one that is MORE important than the other.”

### Results and Discussion

All comparisons resulted in 81 triads with all possible combinations of pairwise choices between motivation types (motives). Thus, in some triads, the same motive could be represented by different statements (of the same motive), so that in a triad IE – ES – SI the statements i1, i2, and i3 could do as interchangeable for I, as well as e1, e2, and e3 for E and s1, s2 and s3 for S. This could lead to conflating effects of between-statements differences (within the same motive) with between-motives differences. Although we used statements strongly associated with their motivation types, but some semantic differences between them could still have place and affect the ordering. Therefore, we analyzed only those triads where the fixed statement presented each motive. For instance, we did analyze the triad i1e1 – e1s1 – i1s1, but not a triad i1e1 – e1s1 – i1s*2*, even though s1 and s2 are of the same motivation type.

Therefore, for each of the 250 participants, we analyzed 27 triads.

We found that the proportion of transitivity violations, i.e., the proportion of triads without transitively ordered motives, was 5% (median 0), ranging from 0 to 44% per participant (Supplementary Table 1). It is important to note that 152 participants (61.8%) did not show transitivity violations at all.

A large proportion of violations seemed to come from a few participants (Supplementary Figure 1). About 60% of the “violators” were found to have 1–3 intransitive triads among 27 (Supplementary Table 1). The distribution of transitivity violations between triads was somewhat uniform and varied from 2 to 9% (median 5%), implying that there were no individual triads provoking violations.

To estimate the percentage of violation variance related to individual differences in participants, we estimated the intraclass correlation coefficient (ICC) in the two-level logistic model (with individuals as first-level and items as second-level). We conducted a multilevel analysis for a base model without any predictors in lme4 package in RStudio. In this case, Random Effect Variance=3.94 and the between-group ICC=0.545. Thus, 55% of the variance can be attributed to the variations across participants, not items.

In general, the violations rate seems to support the hypothesis about the ordinal structure of motivation, at least for these three motivation types and for two levels (“more” or “less”). Morris et al. (2017) reported a 0.04 violation rate even when people ordered attributes of definitely quantitative structures, such as height and weight. In other words, if we have 4% of transitivity violations with undeniably quantitative attributes, then 5% of violations obtained for the motivation attribute can be considered as an argument in favor of the ordinal structure of motivation.

Nevertheless, there is a serious restriction for this argument since a significant part of our sample was not able to preserve transitivity in comparisons. This fact cannot be simply ignored; transitivity violations must be subject to closer scrutiny. Generally, violations can be considered as either random or systematic errors. Would violations have been purely random, they were uniformly distributed over the sample and occurred more frequently. Indeed, the chance of one triad being violated is 0.25 as there are two possible intransitive triads among eight. Moreover, since 60% of the sample has no transitivity violations and since among “violators” the distribution of violations noticeably differed from overall and had a median proportion 0.11 (see Supplementary Table 1), there must be some systematic sources for errors in the remaining participants.

The question is what is the source of these violations? One explanation is that these participants do not clearly distinguish motives. The other is that their motives are inconsistent across situations to which they refer or people change their minds when completing the questionnaires. This is what in decision making area researchers describe with a mixture model of transitive preference (MMTP; Regenwetter et al., 2010, 2011). Both options imply that it is not possible to conclude about the motivation hierarchy for these individuals. Another explanation is that they do have some motivation hierarchy, but levels of their motives were somewhat subjectively close to each other, thereby hampering the ordering. If so, the non-strict transitivity condition would affect these cases differently. Where there was an absence of hierarchy, the proportion of violations must remain unaffected; however, where there was a hierarchy with close levels of some motives, the proportion of violation must decrease because the “equally” option in the non-strict condition would relax the constraints of strict order.

## Study 2

In this study, we aimed to check the transitivity hypothesis under the condition of weak transitivity. Based on some propositions from the literature (Michell, 1998), we could expect relaxing constraints of strict order and, accordingly, decreasing transitivity violations. The logic behind these propositions is that the closer the levels of attributes, the harder they are to order. However, this is only true if people can discern levels of attributes clearly enough to indicate when the levels are equal; otherwise, they face the same issues as with strict ordering, as difficulties of discerning similar levels as “less” vs. “more” will remain the same when discerning similar levels as “less (or more)” vs. “equal.” Therefore, insofar as the observed violations were caused by the closeness of motivation levels, then the violation rate will decrease in the non-strict transitivity condition. Moreover, we can expect a decrease in the proportion of “violators” and the proportion of violations between “violators.” However, if violations are from other sources (e.g., the absence of motivation hierarchy in “violators”), the violation rate will not decrease.

### Method

#### Participants

The responses of 94 university students (mean age 23.1; *SD*=1.5), who completed the questionnaire online and passed the quality control, were included in further analysis.

#### Instruments and Procedure

The questionnaire and procedure were the same as in Study 1, except for two changes. We added the following sentence “If both motives are equally important and you REALLY cannot decide which is more important, select the option ‘Equally important.’” Accordingly, the option “Equally important” was added to each pair of statements.

### Results and Discussion

#### Analysis of Transitivity

We first analyzed whether motivation types were ordered while preserving non-strict transitivity in triads. A triad is transitive, if *I* ⩾ *E*, *E* ⩾ *S*, and *I* ⩾ *S*; otherwise it violates transitivity. With three options (more, less, and equally), the ratio of correct and intransitive patterns for a triad differed from Study 1. Here, there could be 13 “correct” patterns for each triad, where transitivity is preserved, for example, IE IS ES (the first letter points to a dominant motivation in a pair), and 14 “intransitive” patterns, for example, IE SI E=S.

About 60% of participants (*n*=55) were found to have no transitivity violations. Overall, the distribution of the proportion of transitivity violations ranged from 0 to 55% (median 0) per participant and average violation rate 4% (see Supplementary Table 1). This observed rate was quite small, taking into account 4% of violations obtained when people order clearly quantitative attributes (Morris et al., 2017).

The proportion of transitivity violations between triads was somewhat uniform and varied from 2 to 10% with a median of 5% per triad, implying that individual triads were not responsible for the distribution of violations. Indeed, the largest proportion of violations (80%) came from a smaller proportion of the participants (40%; see Supplementary Figure 2). About 70% of the “violators” were found to have only 1–3 intransitive triads among 27 (Supplementary Table 1). In addition, the individual-level ICC for Study 2 was the same as for Study 1 (ICC=0.547, Random Effect Variance=3.982). Therefore, about 55% of the variance is also related to participant differences.

As in Study 1, these results favor the hypothesis regarding the ordinal structure of motivation, but, again, the fact that almost half the participants were not able to preserve transitivity requires closer examination.

Since the violations came from only a smaller proportion of the participants, we can conclude that there must be individual-related specific systematic errors. Since the proportion of violations remains unaffected under the non-strict condition comparing to the strict condition in Study 1, we conclude that the source of intransitivity lies not in similar levels of motives, which are difficult to order, but rather in the inconstant (across situations and time) hierarchy of motives in the “violators.” Some support for this conclusion came from the distribution of the “equally” option.

#### Distribution of the “Equally” Option

The “equally” option was a crucial element in Study 2 because, as is generally assumed, this option may be required when people perceive levels of motives as similar. If this assumption is true, the frequency of the usage of this option indicates to what extent subjectively similar levels would hamper (and could have hampered) the ordering in the strict transitivity condition. About 47% of the participants used the “equally” option at least once; on average, they assigned “equally” to six pairs of motives among 27 (varying from 1 to 18 pairs, median=4). Judging by this rate, the transitivity violations observed in Study 1 could, perhaps, have been caused by the constraints of strict ordering.

Indeed, on the one hand, “equally” option was used mostly by “violators” (84%), meaning that violations and resorting to the “equally” option are associated. Indeed, among “violators,” the correlation between the rate of “equally” in pairwise comparisons and the violation rate was 0.74. Based on this, we can hypothesize that judging motives as “equal” and transitivity violations stemmed from the same source, specifically, from subjectively similar levels of motives. However, on the other hand, as the non-strict ordering did not eliminate violations, it seems that this source cannot be controlled by the “equally” option. It seems the source of violations remains the same in both strict and non-strict conditions; it just moved from one “disputed territory” (more vs. less) to the other (more vs. equal).

## General Discussion and Conclusion

We tested the widespread assumption that motivation has an ordinal structure, checking if transitivity, an axiomatic condition for any ordinal structure, is preserved when people compare different motives (intrinsic, extrinsic, and social) in terms of their importance to learning. The results showed that most participants could preserve transitivity when ordering motives, supporting the underlying assumption.^2^ At the same time, we found that there is a substantial number who could not transitively order motives. This undermines the mainstream use of rating scales in motivation research and requires further scrutiny.

Based on the distribution of the transitivity violations across two experimental conditions, we can conclude that these violations were unlikely caused only by the closeness of motivation levels, because the non-strict ordering did not eliminate violations. Moreover, the strong correlation of the usage of the “equally” category with the violations rate indicates that for the “violators” has the same difficulty: the choice between “more” and “less” is not easier for them than the choice between “equal” or “different.”

We cannot point to sources of such individual intransitivity unambiguously. Several models of intransitivity tested in the decision making research may be applicable here as explanations of individual intransitivity. For example, hypotheses that participants can change their minds during self-reporting or make random errors seem to be relevant explanations (Cavagnaro and Davis-Stober, 2014). In the scope and with the design of this study we cannot test these models, although it must be very promising to bring these more rigorous methods for evaluating transitivity to personality and motivation research, which typically use descriptive statistics and pure sensible criteria for the interpretation of violation frequencies (Michell, 1998; McGrane, 2009; Morris et al., 2017).

Summarizing, we can conclude that rating scales can at best differentiate between distant levels of attributes and for some participants, but by no means provide any scores on the interval scale, such as rational numbers that are generated and presented in typical psychological studies. Our findings demonstrate the need to verify our assumptions about psychological attributes before erecting quantitative constructions on them. Even if such verification is a step backward, we will at least stay on solid ground.

## Data Availability Statement

The raw data supporting the conclusions of this article will be made available by the authors, without undue reservation.

## Ethics Statement

The studies involving human participants were reviewed and approved by Ethics Committee of Pushkin State Russian Language Institute, Moscow, Russia. The patients/participants provided their written informed consent to participate in this study.

## Author Contributions

YT conceived the presented idea, obtained and analyzed the data, and wrote the manuscript’s first draft. KV obtained and analyzed the data and wrote a part of the manuscript. All authors contributed to the article and approved the submitted version.

## Funding

The research was funded by a Russian Government contract; project FZNM-2020-0005, “Transformation of human cognition and communication in the digital era.”

## Conflict of Interest

The authors declare that the research was conducted in the absence of any commercial or financial relationships that could be construed as a potential conflict of interest.

## Publisher’s Note

All claims expressed in this article are solely those of the authors and do not necessarily represent those of their affiliated organizations, or those of the publisher, the editors and the reviewers. Any product that may be evaluated in this article, or claim that may be made by its manufacturer, is not guaranteed or endorsed by the publisher.

## Footnotes

^1^It is not to say that people are supposed to be able to truly define equality between levels. This assumption can be justified if the attribute has quantitative structure, but this is far from our expectations. We regard the “equally” option rather as a room for subjectively similar levels.

^2^Based on the recommendation of the second reviewer, we run the *a priori* procedure (Li et al., 2020) in order to estimate how good the violation rate obtained on our samples approximate the population proportion. At the level of participants, the sample proportions of “non-violators” approximate the population proportions with the precision 0.06 and 0.1, for Study 1 and 2 accordingly. At the level of triads, the sample proportions of transitivity violations approximate the population proportions with the precision 0.012 and 0.02, for Study 1 and 2 accordingly (all estimates are with the confidence level 0.95). These results suggest that we should have used more sample size in Study 2 for a better approximation of the population rate of “non-violators.”
