# Supplementary material for: Transitivity Violations Undermine Rating Scales in Motivation Research
Source: Front Psychol. 2021 Sep 30;12:632991. doi: 10.3389/fpsyg.2021.632991 (PMC8514613; doi:10.3389/fpsyg.2021.632991)
Supplement: Supplementary file 1 [file Data_Sheet_1.PDF]

Supplementary materials

Table 1. The proportion of transitivity violations per participant overall [among violators] and the proportion of non-violators.

Transitivity	Min	Max	M	Median	Individuals without violations	Number of violators with 1-3 violations
Strict n=250 (Study 1)	0.00 [0.04]	0.44 [0.44]	0.05 [0.13]	0 [0.11]	60.8%	61.2%
Weak n=94 (Study 2)	0.00 [0.03]	0.56 [0.56]	0.06 [0.14]	0 [0.11]	58.5%	69.2%

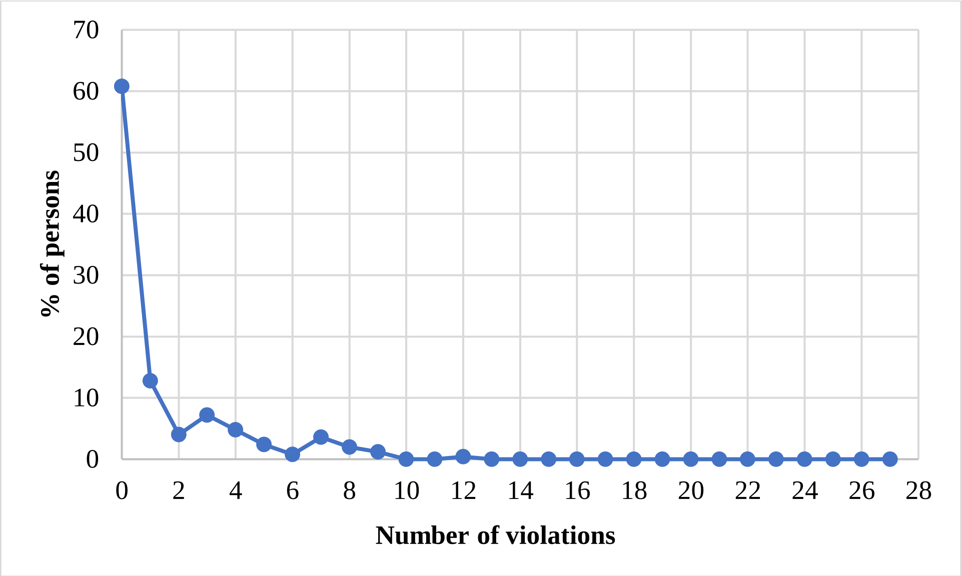

Figure 1. The distribution of transitivity violations among the participants of Study 1.

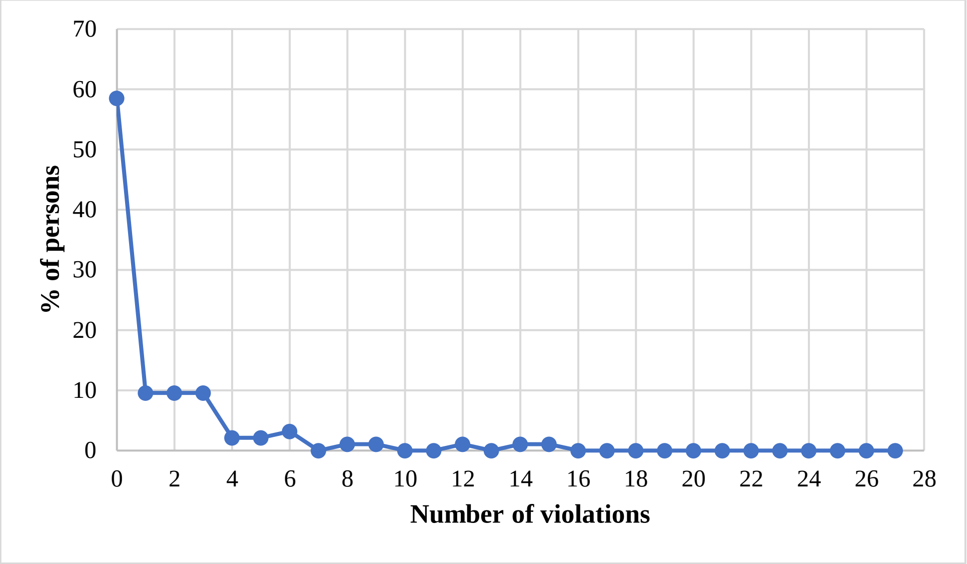

Figure 2. The distribution of transitivity violations among the participants of Study 2.
