# Supplementary material for: Transitivity Violations Undermine Rating Scales in Motivation Research
Source: Front Psychol. 2021 Sep 30;12:632991. doi: 10.3389/fpsyg.2021.632991 (PMC8514613; doi:10.3389/fpsyg.2021.632991)
Supplement: Supplementary file 2 [file Data_Sheet_2.PDF]

Appendix

First of all, I go to my classes...

1) -to meet my friends
   -I am interested in learning

2) -to worry less about my future
   - what we study is important for me

3) - because it has a sense of community
   -to obtained a diploma

4) -to obtained a diploma
   - I am interested in learning

5) - otherwise, I will not be able to find a job
   - what we study is important for me

6) - I am interested in learning
   - otherwise, I will not be able to find a job

7) -to obtained a diploma
   - to meet new people

8) - what we study is important for me
   - to meet new people

9) - to know more about what we study
   -to worry less about my exams

10) - because it has a sense of community
    - what we study is important for me

11) -to worry less about my exams
    -to meet my friends

12) - to know more about what we study
    -to meet my friends

13) - to meet new people
    - I am interested in learning

14) - I am interested in learning
    - because it has a sense of community

15) - because it has a sense of community
    - otherwise, I will not be able to find a job

16) - I am interested in learning
    - to worry less about my exams

17) - what we study is important for me
    -to meet my friends

18) - to meet new people
- to know more about what we study

19) - to know more about what we study
-to obtained a diploma

20) - otherwise, I will not be able to find a job
-to meet my friends

21) - because it has a sense of community
- to worry less about my exams

22) - because it has a sense of community
- to know more about what we study

23) - to know more about what we study
- otherwise, I won't be able to find a job

24) -to obtained a diploma
- what we study is important for me

25) - otherwise, I will not be able to find a job
- to meet new people

26) -to obtained a diploma
-to meet my friends

27) - to worry less about my exams
- to meet new people
